# Novel Insights From In Silico Analysis of Biallelic ALPL (c.1001G/A and c.571G/A) in Two Mennonite Families Leading to Hypophosphatasia

**DOI:** 10.7759/cureus.93139

**Published:** 2025-09-24

**Authors:** Víctor M Salinas-Torres, Rafael A Salinas-Torres, Jesús S Velarde-Felix, Yuriria Rufino-Serralde, Antje Roeniger-Desatnik, Manuel A Villagrán-Luján, Ana B Mata-Martínez, Jorge Ramírez-Zenteno

**Affiliations:** 1 Genomic Medicine, Servicios de Salud del Instituto Mexicano del Seguro Social para el Bienestar, Hospital General de Culiacán, Culiacán, MEX; 2 Faculty of Medicine, Universidad Autónoma de Sinaloa, Culiacán, MEX; 3 Systems and Computing, Instituto Tecnológico de Tijuana, Tijuana, MEX; 4 Faculty of Biology, Universidad Autónoma de Sinaloa, Culiacán, MEX; 5 Rehabilitation Medicine, Integrated Rehabilitation Center Teleton, Chihuahua, Chihuahua, MEX; 6 Orthopaedics, Integrated Rehabilitation Center Teleton, Chihuahua, Chihuahua, MEX; 7 Orthodontics, Integrated Rehabilitation Center Teleton, Chihuahua, Chihuahua, MEX; 8 Genetics, Integrated Rehabilitation Center Teleton, Chihuahua, Chihuahua, MEX

**Keywords:** alpl, genetics, hypophosphatasia, in-silico modeling, mutation

## Abstract

This report aimed to describe two families of Mennonite heritage affected with hypophosphatasia (HPP) and biallelic *ALPL* c.1001G>A/c.571G>A in four individuals. Additionally, we conduct a systematic review of studies considering the above compound heterozygous genotype and present novel insights and evidence inferred from in silico predictions using Ensembl's Variant Effect Predictor (VEP) platform and STRING protein-protein interaction (PPI) network analysis to explore these genetic variations and plausible interacting pathways for the disorder that remain for consideration in future studies. Intrafamilial and interfamilial variability of phenotypes was observed in the four patients affected with the identical *ALPL* c.1001G>A/c.571G>A mutation. In contrast, in the seven unaffected family members, a specific genotype was not available. Seven eligible studies exploring *ALPL* c.1001G>A/c.571G>A were identified, and significant heterogeneity (*P* < 0.05) was observed across four studies. Ensembl VEP inferred a dual effect for rs121918007 and rs121918009, involving 17 variants located in the exome and four classified as non-coding associated with all HPP presentations, as well as serum alkaline phosphatase levels, choline phosphate levels, osteogenesis imperfecta, and inborn genetic diseases. PPI network modeling predicted 10 genes (*PTS, NBPF3, SLC30A7, TPK1, NTPCR, BGLAP, RUNX2, ENPP1, SLC30A6, GCH1*) interacting with *ALPL*, highlighting their potential impact on bone formation and homeostasis, metabolism, and gene expression. These results may shed light on HPP variability by disrupting key metabolic and transcriptional pathways and provide a comprehensive view of their functional relevance, which suggests a complex genetic etiology for HPP.

## Introduction

Hypophosphatasia (HPP; OMIM 241500, 241510, 146300) is a rare genetic disorder characterized mainly by defective bone mineralization with or without root-intact tooth loss due to inactivating variants in the *ALPL* gene, encoding the tissue-nonspecific alkaline phosphatase enzyme [1]. Clinical subtypes of HPP include perinatal severe (characterized by restrictive lung disease and high mortality), perinatal benign (prenatal skeletal manifestations that slowly resolve into one of the milder forms), infantile (onset between birth and age six months of clinical features of rickets without elevated serum ALP activity), severe childhood/juvenile (variable presenting features progressing to rickets), mild childhood (present later in childhood without rachitic disease, low bone mineral density for age, increased risk of fracture, and premature loss of primary teeth with intact roots), adult (characterized by osteomalacia, stress fractures, or pseudofractures), and odontohypophosphatasia (characterized by premature exfoliation of primary teeth and/or severe dental caries without skeletal manifestations) [1,2].

The inheritance pattern heterogeneity caused either by homozygous, compound heterozygous, or heterozygous mutation in *ALPL*, along with its marked variability in clinical expression, makes the diagnosis of HPP often missed or delayed [2]. Furthermore, over 400 different disease-causing variants in the *ALPL* gene have been identified, contributing to extremely variable presentations within and between families, in both children and adults [1,2]. Although infantile rickets, pathologic fractures, premature loss of deciduous teeth, and abnormalities in serum pyridoxal 5'-phosphate and serum unfractionated alkaline phosphatase activity are suggestive of HPP, other factors also delay the HPP diagnosis, such as limited awareness of HPP in the medical community or incomplete penetrance of dominant forms; hence, achieving a precise diagnosis of HPP is challenging for suspected patients [2].

In this sense, given the genotype-phenotype variability in HPP, the identification of additional alleles at a locus may help uncover further insights for a causal gene in the region. Thus, determining the precise implications of the genetic variation considering systematic approaches and in silico analysis is a reliable tool to address the above issues, unraveling causes for Mendelian diseases and pinpointing a more complex genetic etiology; hence, enhancing our ability to understand biological mechanisms related to disease genes within the human network [3].

Here, we report four patients affected with HPP who had biallelic *ALPL* mutations c.1001G>A (rs121918007) and c.571G>A (rs121918009) from two nuclear families of Mennonite heritage. We also conduct a systematic review of the above compound heterozygous genotype, considering 29 reported cases [4-10], and present novel insights and evidence inferred from in silico predictions to explore these genetic variations and plausible interacting pathways for the disorder that remain for consideration in future studies.

## Case presentation

There were 11 family members from two nuclear families of Mennonite heritage, four of whom exhibit HPP-related signs and symptoms and the biallelic *ALPL* mutation c.1001G>A/c.571G>A, whereas seven family members were unaffected, and a specific genotype was not available. Table 1 summarizes the four patients affected with HPP.

**Table 1:** Summary of clinical and laboratory findings in hypophosphatasia patients ^a^Based on the World Health Organization child growth standards reference ^b^Based on the USA combined National Health and Nutrition Examination Survey/Lunar reference ^†^Values in parentheses are the reference serum values BMI, body mass index; AP, alkaline phosphatase; PLP, pyridoxine 5-pyrophosphate; Ca, calcium; P, phosphorus; DXA, dual-energy x-ray absorptiometry scan

Feature	Family One		Family Two	
	Case one	Case two	Case three	Case four
Age in years	13	7	10	6
Sex	Female	Female	Female	Female
BMI, kg/m^2^ (z-score)^a^	20.6 (0)	14.9 (−1)	13.3 (−2)	14.0 (−1)
Early nontraumatic loss of primary teeth	Yes	Yes	Yes	Yes
Infantile rickets	Yes	Yes	Yes	Yes
Linear growth failure	Yes	Yes	Yes	Yes
Gross motor delay	Yes	Yes	Yes	Yes
Craniosynostosis	No	No	Yes	No
Nephrocalcinosis	No	No	No	Yes
Vitamin B6-responsive seizures	No	No	No	No
AP(141-460 U/L)^†^	11258.7	8664.2	9015	8704
PLP (16.0-64.0 µg/L)^†^	15.4	20.3	26.3	33.2
Ca (8.3-9.8 mg/dL)^†^	6.6	8.3	9.9	8.7
P (4.0-5.2 mg/dL)^†^	2.8	5.0	6.0	5.4
DXA, g/cm^2 ^(z-score)^b^	0.880 (−0.9)	0.664 (−0.5)	0.594 (−2.1)	0.590 (−1.7)
*ALPL* variants	c.1001G>A (p.Gly334Asp)/c.571G>A (p.Glu191Lys)	c.1001G>A (p.Gly334Asp)/c.571G>A (p.Glu191Lys)	c.1001G>A (p.Gly334Asp)/c.571G>A (p.Glu191Lys)	c.1001G>A (p.Gly334Asp)/c.571G>A (p.Glu191Lys)

Family one

The proband (case one) was the first daughter of healthy, non-consanguineous parents aged 37 (mother) and 38 (father), both of Mennonite heritage. She presented with signs and symptoms of confirmed HPP at 13 years old. Her history included multiple decayed teeth with premature loss of central incisors and infantile rickets characterized by growth failure, hypotonia, lax ligaments (genu varum), flail chest, costochondral enlargement, scoliosis, flared metaphyses, and bowed long bones. During childhood, she required a tonsillectomy and a spinal fusion surgery due to a curve magnitude of 60 degrees at seven and 12 years old, respectively. There were no additional dysmorphic features, and the rest of the physical examination, as well as the neurologic, cardiopulmonary, hepatic, and renal assessments, was unremarkable.

An extensive metabolic workup, including neonatal metabolic biomarkers and endocrinological investigations, was within the reference values; however, abnormalities of serum alkaline phosphatase (AP), pyridoxine 5-pyrophosphate (PLP); calcium (Ca), and phosphorus (P) were identified. The dual-energy X-ray absorptiometry (DXA) screening showed no indication of osteoporosis. Additionally, gene-targeted sequence analysis through the Invitae HPP panel (>99% analytical sensitivity and specificity for single nucleotide variants, insertions and deletions <15 bp in length, and exon-level deletions and duplications), including the *ALPL*,* CLCN5, CTNS, CYP27B1, CYP2R1, DMP1, ENPP1, FAH, FAM20C, FGF23, FGFR1, GNAS, OCRL, PHEX, SLC34A1, SLC34A3, *and* VDR* genes, detected two pathogenic variants in *ALPL *(c.1001G>A (p.Gly334Asp) and c.571G>A (p.Glu191Lys)) reported on October 20, 2021.

According to the Invitae HPP panel, the c.1001G>A (p.Gly334Asp) variant was classified as pathogenic for the following reasons: (i) the glycine residue was moderately conserved, and there was a moderate physicochemical difference between glycine and aspartic acid; (ii) the variant was not present in population databases (ExAC no frequency); (iii) missense changes were observed in individuals with HPP [4]; (iv) there was an entry variation in ClinVar (ID: 13672); (v) algorithms developed to predict the effect of missense changes on protein structure and function were either unavailable or did not agree on the potential impact of the missense change (SIFT: "Not Available"; PolyPhen-2: "Probably Damaging"; Align-GVGD: "Not Available"); and (vi) experimental studies showed that the missense change affected *ALPL* function [4].

The c.571G>A (p.Glu191Lys) variant was classified as pathogenic for the following reasons: (i) the glutamic acid residue was moderately conserved, and there was a small physicochemical difference between glutamic acid and lysine; (ii) the variant was present in population databases (ExAC 2.0%); (iii) missense change were observed in individuals with HPP [4]; (iv) there was an entry variation in ClinVar (ID: 13670); (v) algorithms developed to predict the effect of missense changes on protein structure and function were either unavailable or did not agree on the potential impact of the missense change (SIFT: "Not Available"; PolyPhen-2: "Probably Damaging"; Align-GVGD: "Not Available"); and (vi) experimental studies showed that the missense change affected *ALPL* function [4].

Treatment with asfotase alfa (a bone-targeted enzyme replacement therapy designed to address the deficient alkaline phosphatase) began in October 2022, showing improvement of chronic joint and muscle pain, muscle weakness, and severe limitations in activities of daily living. The family history was negative for pregnancy losses or early childhood death but positive for her sister diagnosed with HPP at seven years old (case two), while her nine-year-old brother showed no signs or symptoms related to HPP.

Case two presented multiple decayed teeth with premature loss of central incisors and infantile rickets characterized by growth failure, hypotonia, lax ligaments, flail chest, costochondral enlargement, flared metaphyses, and bowed long bones. At three years old, she had a history of bilateral epiphysiodesis due to genu varum and fractures in her left femur. Abnormal serum AP levels were detected, and additional gene-targeted sequence analysis through the Invitae HPP panel on October 20, 2021, detected two pathogenic variants in *ALPL* (c.1001G>A (p.Gly334Asp) and c.571G>A (p.Glu191Lys)). DXA screening showed no indication of osteoporosis. Further physical, laboratory, and imaging examinations were unremarkable. Limitations in activities of daily living decreased due to treatment with asfotase alfa, which began in October 2022.

Family two

The proband (case three) was the second daughter of healthy, non-consanguineous parents aged 31 (mother) and 44 (father), both of Mennonite heritage. She presented with signs and symptoms of confirmed HPP at 10 years old. Her history included multiple decayed teeth without premature loss of deciduous teeth and infantile rickets characterized by growth failure, frontal-parietal exostosis, craniosynostosis, hypotonia, lax ligaments (genu varum), flail chest, costochondral enlargement, scoliosis, flared metaphyses, and bowed long bones. There were no additional dysmorphic features and further neurologic, cardiopulmonary, hepatic, and renal assessments showed no abnormalities.

Extensive metabolic workup, including neonatal metabolic biomarkers and endocrinological investigations, was within the reference values, except for high serum values of AP, Ca, and P. Furthermore, DXA screening was suggestive of osteoporosis and gene-targeted sequence analysis through the Invitae HPP panel on January 23, 2023, detected two pathogenic variants in *ALPL* (c.1001G>A (p.Gly334Asp) and c.571G>A (p.Glu191Lys)). In February 2023, infusion with asfotase alfa began and the patient's functional status improved considerably. The family history was negative for pregnancy losses or early childhood death but positive for her sister diagnosed with HPP at six years old (case four), whereas her 17-year-old sister and his two-year-old brother showed no signs or symptoms related to HPP.

Case four presented multiple decayed teeth with premature loss of central incisors and infantile rickets characterized by growth failure, hypotonia, lax ligaments (genu varum), flail chest, costochondral enlargement, scoliosis, flared metaphyses, and bowed long bones. At six years old, she had a history of genu varum, pes planus, and nephrocalcinosis. High serum AP levels were detected, and gene-targeted sequence analysis through the Invitae HPP panel on January 23, 2023, detected two pathogenic variants in *ALPL* (c.1001G>A (p.Gly334Asp) and c.571G>A (p.Glu191Lys)). DXA screening was suggestive of osteoporosis. Further physical, laboratory, and imaging examinations were unremarkable. After asfotase alfa was initiated in February 2023, the patient experienced clinical improvement and reduced the use of assistive devices.

## Discussion

Literature search of biallelic mutation *ALPL* (c.1001G>A/c.571G>A)

Published studies were identified using the Web of Science and PubMed/MEDLINE databases. A systematic review of the literature was performed using the checklist for the Preferred Reporting Items for Systematic Reviews and Meta-Analyses (PRISMA). The search terms were (hypophosphatasia), (ALPL), and (polymorphism or gene or allele or SNP). No restriction was applied to language, geographical location, or study design in the literature search process. The search was conducted from inception to May 31, 2025.

Observational studies with quantitative data that examined *ALPL* mutations were considered. Inclusion criteria were articles reporting original data about biallelic *ALPL* mutation (c.1001G>A/c.571G>A) on HPP. Studies were excluded if they met the following exclusion criteria: (1) lack of data about biallelic *ALPL* mutation (c.1001G>A/c.571G>A); (2) repeated studies; and (3) animal or experimental studies.

Two reviewers (V.M.S.T. and R.A.S.T.) independently evaluated the eligibility criteria by looking through all titles and abstracts of the identified studies and also extracted the data using a standardized form. From the selected articles, a review of the reference lists was done to identify additional relevant publications. For the included studies, the following data were collected: first author, year of publication, population, number of confirmed *ALPL* mutations, and *ALPL* genotype distribution as follows: number of compound heterozygous genotypes with c.1001G>A/c.571G>A, number of any compound heterozygous genotypes without c.1001G>A/c.571G>A, number of any homozygous genotypes, and number of any heterozygous genotypes. These data were computerized separately by each reviewer as well as validated to identify and solve disagreements.

Published data regarding *ALPL* mutations on HPP were deduced from the reported data and presented in numbers and percentages. Except for the compound heterozygous genotype c.1001G>A/c.571G>A, the rest of the genotypes were combined for the analyses due to the small case counts across studies. The frequency of the above ALPL genotypes was compared using contingency tables for each study (Fisher's exact test, accounting for heterogeneity). All statistical analyses were performed using the IBM SPSS Statistics for Windows, Version 21 (Released 2012; IBM Corp., Armonk, New York). All P-values presented were two-tailed with a significance level of <0.05.

Heterogeneity in ALPL c.1001G>A/c.571G>A

**Figure 1:**
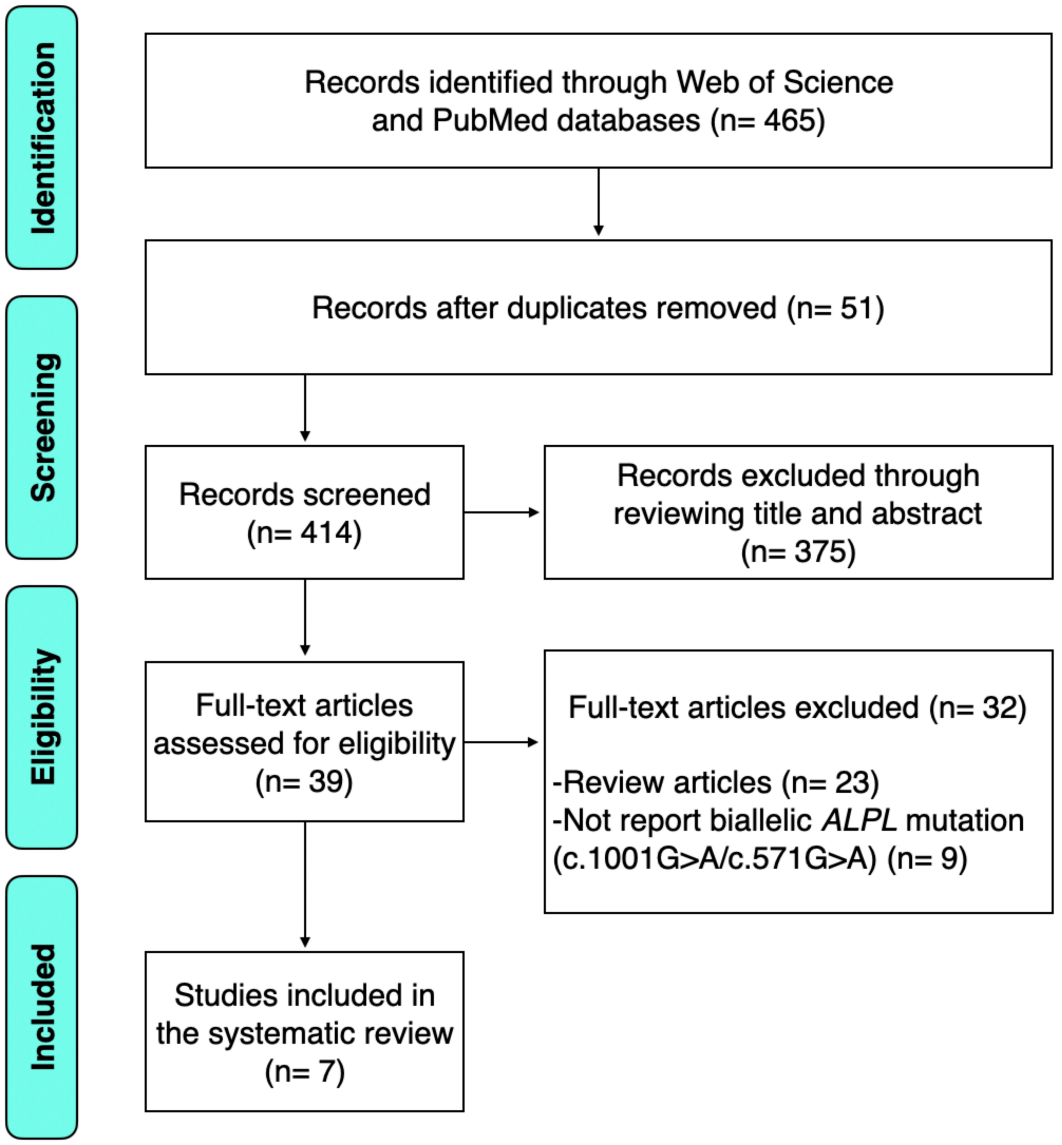
The PRISMA flow diagram PRISMA, Preferred Reporting Items for Systematic Reviews and Meta-Analyses

The initial literature search yielded 465 articles from Web of Science and PubMed/MEDLINE databases. After removing repeated publications, 414 studies were left to be screened. Titles and abstracts from all studies were screened, and 375 studies were excluded for irrelevance. The remaining 39 studies were screened by reading the full text, and seven studies were identified that met the inclusion criteria [4-10]. A flow diagram of the selection process and search results is shown in Figure 1.

The following table summarizes the *ALPL* genotype distribution from seven studies reporting c.1001G>A/c.571G>A (Table 2). A comparative analysis of the frequency of *ALPL* genotypes revealed statistical significance (P < 0.05) across four studies [4,6,7,9], whereas no indication of significant heterogeneity was observed for *ALPL* c.1001G>A/c.571G>A in the other three independent studies [5,8,10].

**Table 2:** ALPL genotype distribution from the included studies ^a^Caucasian population ^b^North America population ^c^Mixed population A, compound heterozygous genotypes excluding c.1001G>A/c.571G>A; B, homozygous genotypes; C, heterozygous genotypes. A, B, and C were combined for the analyses using Fisher's exact test

Author (Year)	Study Design	Confirmed *ALPL* Mutation, n=1423	Genotypes, Number (Frequency)				*P*-value
			Genotype c.1001G>A/c.571G>A, n=29	A, n=420	B, n=102	C, n=872	
Hofmann et al. (2014)^a ^[4]	Family-based	5	2 (0.4)	0	0	3 (0.6)	0.003
Whyte et al. (2015)^b ^[5]	Cohort	105	3 (0.02)	39 (0.3)	0	63 (0.6)	0.468
Huggins et al. (2020)^a ^[6]	Family-based	25	3 (0.1)	6 (0.2)	0	16 (0.6)	0.013
Mornet et al. (2021)^c ^[7]	Cohort	424	3 (0.007)	187 (0.4)	68 (0.1)	166 (0.3)	0.022
Kishnani et al. (2021)^c ^[8]	Cohort	44	3 (0.06)	26 (0.5)	1 (0.02)	14 (0.3)	0.057
Rockman-Greenberg et al. (2022)^a ^[9]	Case series	6	2 (0.3)	2 (0.3)	0	2 (0.3)	0.005
Kishnani et al. (2024)^c ^[10]	Cohort	814	13 (0.01)	160 (0.1)	33 (0.04)	608 (0.7)	0.188

In silico analyses

For the in silico analysis inference, the Ensembl's Variant Effect Predictor (VEP) bioinformatics platform (https://www.ensembl.org/Homo_sapiens/Info/Index, accessed on June 7, 2025) was employed to annotate *ALPL* genetic variants rs121918007 (c.1001G>A (p.Gly334Asp)) and rs121918009 (c.571G>A (p.Glu191Lys)). The resulting consequence type calls allow for classifying them into categories, such as missense and regulatory region variants, and assessing the potential pathogenicity of the variants [11]. The analysis was based on Ensembl platform genomics data inferred from the human genome annotation GRCh38 [12]. Furthermore, a protein-protein interaction (PPI) network associated with the *ALPL* gene, including physical, functional, and biological processes, was created using the String database version 12.0 (https://string-db.org, accessed on June 7, 2025). A gene-gene pairwise network detected with a medium confidence score (0.400) was constructed using two or more PPI, including co-expression, protein homology, gene neighborhood, curated databases, textmining, or experimentally determined data sets from the network analysis. The STRING database includes a "hierarchical view" as a structure of the most significant classifications and ontologies of the human genes and E-value statistics (P-values of less than 0.05 adjusted to false discovery rate (FDR)) [13].

VEP Analysis

Variants in the *ALPL* gene, rs121918007 (c.1001G>A (p.Gly334Asp)) and rs121918009 (c.571G>A (p.Glu191Lys)), have been classified with a dual effect (influencing the protein-coding sequence and regulatory elements of the gene) in 17 coding and four non-coding regions of the alternative transcripts of the gene. Table 3 summarizes the in silico VEP analysis of these genetic variants.

**Table 3:** Consequence and potential pathogenicity of ALPL genetic variants rs121918007 and rs121918009 inferred from in silico variant effect predictor analysis *Impact rating and consequence details extracted from Ensembl Chr, chromosome; HGVS, Human Genome Variation Society; AF, allelic frequency in genome aggregation database exomes combined population; SG, stop gained; CADD, combined annotation dependent depletion; DANN, deep neural network; SIFT, sorting intolerant from tolerant; PolyPhen, polymorphism phenotyping; MetaSVM, meta support vector machine; ClinPred, identifying disease-relevant nonsynonymous variants; BLOSUM62, Blocks Substitution Matrix 62; RRV, regulatory region variant (non-coding transcript variant)

Chr	Gene	Marker	HGVS Nomenclature	Impact*	Consequence Details*	AF
1	ALPL	rs121918007	ENST00000374832.5:c.571G>T ENSP00000363965.1:p.Glu191Ter	High	SG; CADD: likely deleterious; DANN: 0.997	−
		rs121918007	ENST00000374840.8:c.571G>T ENSP00000363973.3:p.Glu191Ter	High	SG; CADD: likely deleterious; DANN: 0.997	−
		rs121918007	ENST00000539907.5:c.340G>T ENSP00000437674.1:p.Glu114Ter	High	SG; CADD: likely deleterious; DANN: 0.997	−
		rs121918007	ENST00000540617.5:c.406G>T ENSP00000442672.1:p.Glu136Ter	High	SG; CADD: likely deleterious; DANN: 0.997	−
		rs121918007	ENST00000374832.5:c.571G>A ENSP00000363965.1:p.Glu191Lys	Moderate	Missense; SIFT: deleterious; PolyPhen: possibly damaging; MetaSVM: damaging; ClinPred: tolerant; CADD: likely deleterious; DANN: 0.999; BLOSUM62: 1	0.001
		rs121918007	ENST00000374832.5:c.571G>C ENSP00000363965.1:p.Glu191Gln	Moderate	Missense; SIFT: deleterious; PolyPhen: possibly damaging; MetaSVM: damaging; ClinPred: damaging; CADD: likely deleterious; DANN: 0.995; BLOSUM62: 2	2.05e-6
		rs121918007	ENST00000374840.8:c.571G>A ENSP00000363973.3:p.Glu191Lys	Moderate	Missense; SIFT: deleterious; PolyPhen: possibly damaging; MetaSVM: damaging; ClinPred: tolerant; CADD: likely deleterious; DANN: 0.999; BLOSUM62: 1	0.001
		rs121918007	ENST00000374840.8:c.571G>C ENSP00000363973.3:p.Glu191Gln	Moderate	Missense; SIFT: deleterious; PolyPhen: possibly damaging; MetaSVM: damaging; ClinPred: damaging; CADD: likely deleterious; DANN: 0.995; BLOSUM62: 2	2.05e-6
		rs121918007	ENST00000539907.5:c.340G>A ENSP00000437674.1:p.Glu114Lys	Moderate	Missense; SIFT: deleterious; PolyPhen: benign; MetaSVM: damaging; ClinPred: tolerant; CADD: likely deleterious; DANN: 0.999; BLOSUM62: 1	0.001
		rs121918007	ENST00000539907.5:c.340G>C ENSP00000437674.1:p.Glu114Gln	Moderate	Missense; SIFT: deleterious; PolyPhen: benign; MetaSVM: damaging; ClinPred: damaging; CADD: likely deleterious; DANN: 0.995; BLOSUM62: 2	2.05e-6
		rs121918007	ENST00000540617.5:c.406G>A ENSP00000442672.1:p.Glu136Lys	Moderate	Missense; SIFT: deleterious low confidence; PolyPhen: possibly damaging; MetaSVM: damaging; ClinPred: tolerant; CADD: likely deleterious; DANN: 0.999; BLOSUM62: 1	0.001
		rs121918007	ENST00000540617.5:c.406G>C ENSP00000442672.1:p.Glu136Gln	Moderate	Missense; SIFT: deleterious low confidence; PolyPhen: possibly damaging; MetaSVM: damaging; ClinPred: damaging; CADD: likely deleterious; DANN: 0.995; BLOSUM62: 2	2.05e-6
		rs121918007	ENST00000468526.1:n.631G>A	Modifier	RRV; CADD: likely deleterious	0.001
		rs121918007	ENST00000468526.1:n.631G>C	Modifier	RRV; CADD: likely deleterious	2.05e-6
		rs121918007	ENST00000468526.1:n.631G>T	Modifier	RRV; CADD: likely deleterious	−
		rs121918009	ENST00000374830.2:c.77G>A ENSP00000363963.2:p.Gly26Asp	Moderate	Missense; SIFT: tolerated; PolyPhen: possibly damaging; MetaSVM: damaging; ClinPred: damaging; CADD: likely deleterious; DANN: 0.996; BLOSUM62: −1	1.36e-6
		rs121918009	ENST00000374832.5:c.1001G>A ENSP00000363965.1:p.Gly334Asp	Moderate	Missense; SIFT: deleterious; PolyPhen: possibly damaging; MetaSVM: damaging; ClinPred: damaging; CADD: likely deleterious; DANN: 0.996; BLOSUM62: −1	1.36e-6
		rs121918009	ENST00000374840.8:c.1001G>A ENSP00000363973.3:p.Gly334Asp	Moderate	Missense; SIFT: deleterious; PolyPhen: possibly damaging; MetaSVM: damaging; ClinPred: damaging; CADD: likely deleterious; DANN: 0.996; BLOSUM62: −1	1.36e-6
		rs121918009	ENST00000539907.5:c.770G>A ENSP00000437674.1:p.Gly257Asp	Moderate	Missense; SIFT: deleterious; PolyPhen: possibly damaging; MetaSVM: damaging; ClinPred: damaging; CADD: likely deleterious; DANN: 0.996; BLOSUM62: −1	1.36e-6
		rs121918009	ENST00000540617.5:c.836G>A ENSP00000442672.1:p.Gly279Asp	Moderate	Missense; SIFT: deleterious low confidence; PolyPhen: possibly damaging; MetaSVM: damaging; ClinPred: damaging; CADD: likely deleterious; DANN: 0.996; BLOSUM62: −1	1.36e-6
		rs121918009	ENST00000374829.2: n.270G>A	Modifier	RRV; CADD: likely deleterious	1.36e-6

Protein-Protein Interaction Network Analysis

**Figure 2:**
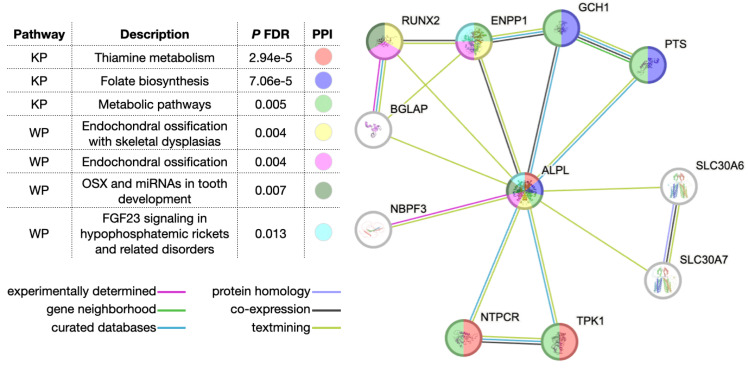
Network modeling from protein-protein interactions showing connectivity directly and via partners with ALPL as well as top associated metabolic and transcriptional pathways KP, Kyoto Encyclopedia of Genes and Genomes Pathways; WP, WikiPathways; FDR, false discovery rate; PPI, protein-protein interaction

The STRING PPI interaction network analysis identified 10 intersecting genes (*PTS*, *NBPF3*, *SLC30A7*, *TPK1*, *NTPCR*, *BGLAP*, *RUNX2*, *ENPP1*, *SLC30A6*, *GCH1*) showing high connectivity via several partners and were closely related to seven top pathways (Figure 2). Thiamine metabolism was the most significantly enriched pathway (*ALPL*, *TPK1*, *NTPCR*, *P* FDR 2.94 × 10^-5^).

ALPL-Associated Genes and Functional Implications From In Silico Analysis

The above analyses enabled the identification of overrepresented biological processes, molecular functions, and pathways and provided novel insights into how the *ALPL* gene, and by extension the variants, might contribute to HPP variability through alterations in pathways related to bone formation and homeostasis, metabolism, and gene expression (Table 4).

**Table 4:** ALPL-associated genes and functional implications summary inferred from protein-protein interaction analysis *Top biological processes and molecular functions extracted from GeneCards

Gene	Location	Functional Implications*
ALPL	1p36.12	Skeletal mineralization, adaptive thermogenesis, skeletal system development, osteoblast differentiation, reproduction, chromatin remodeling
PTS	11q23.1	Biosynthesis of tetrahydrobiopterin, amino acid metabolic process, and central nervous system development
NBPF3	1p36.12	No data available (cellular component located in cytoplasm)
SLC30A7	1p21.2	Zinc ion homeostasis (regulates the activation and folding of alkaline phosphatases), monoatomic ion and cation transport, transmembrane transport
TPK1	7q35	Thiamine metabolic process, biosynthesis of thiamine pyrophosphate to pyrimidines metabolism, regulates pyruvate oxidation and lipogenesis
NTPCR	1q42.2	Nucleotide phosphatase activity towards ATP, GTP, CTP, TTP, and UTP, chromatin remodeling and looping
BGLAP	1q22	Negative regulator of bone formation, skeletal system development, ossification, osteoblast differentiation and development, positive regulation of neurotransmitter secretion
RUNX2	6p21.1	Skeletal system development, ossification, osteoblast differentiation, endochondral ossification, osteoblast fate commitment
ENPP1	6q23.2	Generation of precursor metabolites and energy, phosphate-containing compound metabolic process, immune response, nucleoside triphosphate catabolic process, gene expression
SLC30A6	2p22.3	Contributes to zinc ion homeostasis (activation and folding of alkaline phosphatases), monoatomic cation transport, transmembrane transport, and zinc ion import into the Golgi lumen
GCH1	14q22.2	Tetrahydrobiopterin biosynthetic process, nitric oxide biosynthetic process, regulation of blood pressure and heart rate, negative regulation of cardiac muscle cell apoptotic process

In this report, we describe four patients diagnosed with HPP based on clinical criteria [2] and carrying the pathogenic biallelic *ALPL* mutations c.1001G>A and c.571G>A from two families of Mennonite heritage in Mexico (Table 1). According to our systematic review of the literature, 29 cases present the above compound heterozygous mutation [4-10], and comparative statistical analysis reached statistical significance (*P* < 0.05), which suggests heterogeneity (Table 2). Furthermore, this report employed in silico analyses for *ALPL* (rs121918007 and rs121918009), revealing 17 variants located in the exome and four classified as non-coding (Table 3), as well as a significant overrepresentation of pathways and functional processes related to bone formation and homeostasis, metabolism, and gene expression (Figure 1, Table 4). Together, our results may shed light on HPP pathophysiology, disrupting key metabolic pathways and providing a comprehensive view of their functional relevance in the context of HPP variability and development.

Our observations also confirm the intrafamilial and interfamilial variability of phenotypes that can be observed in patients with an identical genetic compound heterozygous phenotype [1,2]. Interestingly, there was no history of neonatal death in these nuclear families nor HPP-related signs or symptoms in the parents and non-affected siblings (unknown genotype). While not confirmed and based on the extensive allelic heterogeneity in *ALPL* [5,7,8,10], it might be presumed that these unaffected family members may show a heterozygous mutation or slightly reduced serum AP activity [4,6,9]. Yet, it must be noted that the variable nature of the incomplete penetrance of dominant HPP, whereas carriers of a dominant-negative mutation may develop relevant clinical problems in later life. In this report, the lack of mutational analyses in the unaffected family members prevents us from assigning their phenotype to a specific genotype.

It is also possible that additional genetic, epigenetic, or environmental factors may explain the clinical variability. Particularly, intronic mutations or polymorphisms in the *ALPL* or other possibly relevant genes might also have escaped notice. In this report, we present novel insights and evidence inferred from in silico predictions in *ALPL* (rs121918007 and rs121918009) to fill a critical knowledge gap on HPP variability (Table 3). According to Ensembl VEP [11], we found 15 predicted consequences in *ALPL* rs121918007, detailed as stop gained (high impact) in four variants, missense (moderate impact) in eight variants, and regulatory region variant (non-coding transcript variant, modifier impact) in three variants [11]. Alleles T, C, and A of *ALPL* rs121918007 are associated with adult HPP; meanwhile, allele A is also related to childhood, infantile, and odontohypophosphatasia presentations, serum alkaline phosphatase levels, choline phosphate levels, osteogenesis imperfecta, and inborn genetic diseases [12,14-17]. Regarding *ALPL* rs121918009, predicted consequences were detailed as missense (moderate impact) in five variants and a regulatory region variant (non-coding transcript variant, modifier impact) in one variant [11]. Allele A of *ALPL* (rs121918009) is associated with infantile and adult HPP, *ALPL*-related disorders, and osteogenesis imperfecta [12,18].

These results align with the understanding of the intricacies of genetic variations and clinical heterogeneity arising from allelic heterogeneity in HPP [19], as most variants in this report are located in coding regions, compared to non-coding sequences associated with HPP. The latter might reflect different underlying genetic etiologies, adding novel components for this Mendelian disorder and pinpointing a more complex genetic etiology [3,20]. In this sense, the products of *ALPL*-associated genes in this report involve a variety of functions, such as transcriptional factors and regulators, skeletal system development, metabolism, remodeling DNA, and ionic homeostasis (Table 4). To address these associated genes, we individually discuss their implications for HPP.

*PTS* (6-Pyruvoyltetrahydropterin Synthase) and *GCH1* (GTP Cyclohydrolase 1) are involved in the biosynthesis of tetrahydrobiopterin (BH4), an essential cofactor of aromatic amino acid hydroxylases, including enzymes implicated in serotonin biosynthesis and nitric oxide synthase activity. Disorders associated with *PTS* and *GCH1* include phenylketonuria, abdominal obesity-metabolic syndrome, limb-girdle muscular dystrophy, Opitz-Kaveggia syndrome, dystonia, epilepsy, and autism spectrum disorder [21]. PPI analysis showed connectivity directly and via partners with *ALPL* interacting in the folate biosynthesis and metabolic pathways (Figure 1). Functional annotation and Human Phenotype Ontology (HPO) implication of *PTS* and *GCH1* related to HPP comprise heel bone mineral density (rs3819331, rs17127816) [21,22], intellectual disability, global developmental delay, seizure, hypotonia, recurrent fever, scoliosis, and talipes equinovarus [21,23].

*NBPF3* (Neuroblastoma Breakpoint Family Member 3) is a member of the neuroblastoma breakpoint family, which consists of dozens of recently duplicated genes primarily located in segmental duplications on human chromosome 1 [21]. PPI analysis showed experimentally determined connectivity directly with *ALPL* (Figure 1). HPO annotations associated with *NBPF3* are unknown; however, top phenotypes from GWAS include alkaline phosphatase measurement (rs10733029, rs10917021, rs115239632, rs116410304, rs12033776, rs149262245, rs16825415, rs1697405, rs1780324, rs1827293, rs1976403, rs2242420, rs2282713, rs4654748, rs558462412, rs60515836, rs6680628, rs67730681, rs71636972, rs72657141, rs72872292, rs74614333, rs75394487, rs78787495, rs80320018) [21,22]. *NBPF3*-related disorders involve neuroblastoma, Diamond-Blackfan anemia 11, schizophrenia, vitamin B6-dependent epilepsy (early-onset), and microcephaly [21].

*SLC30A7* (Solute Carrier Family 30 Member 7) and *SLC30A6* (Solute Carrier Family 30 Member 6) contribute to zinc ion homeostasis from the cytosol into the lumen of organelles (Golgi and vesicles) along the secretory pathway and regulate the activation and folding of enzymes like alkaline phosphatases. Disorders linked to these genes include Ziegler-Huang syndrome, a neurodevelopmental disorder characterized by short stature, a prominent forehead, and feeding difficulties (NEDSSF), Joubert syndrome, hypermanganesemia with dystonia, and Ehlers-Danlos syndrome [21]. PPI analysis showed connectivity directly and via partners with *ALPL* (Figure 1). The functional annotation and HPO implications of SLC30A7 related to HPP include testosterone measurement (rs7532171) [21,22], intrauterine growth retardation, growth delay, and delayed skeletal maturation [21,23]. No HPO data were available for *SLC30A6*; however, phenotypes from GWAS include testosterone measurement and bone density (rs113017476, rs72796891) [21,22].

Further direct and via partners PPI were observed between *TPK1* (thiamin pyrophosphokinase 1) and *NTPCR* (nucleoside triphosphatase, cancer-related) and *ALPL* interacting in the thiamine metabolism and metabolic pathways (Figure 1). On the one hand, *TPK1* acts as a homodimer and catalyzes the conversion of thiamine-to-thiamine pyrophosphate, a cofactor for some enzymes of the glycolytic and energy production pathways (oxidation and lipogenesis). *TPK1*-related disorders comprise thiamine metabolism dysfunction syndrome, abdominal obesity-metabolic syndrome, limb-girdle muscular dystrophy, Opitz-Kaveggia syndrome, dystonia, epilepsy, and autism spectrum disorder [21]. Functional annotation and HPO implications of *TPK1* related to HPP involve bone density (rs185266914), metabolic disease (rs73164732) [21,22], global developmental delay, seizure, hypotonia, and microcephaly [21,23]. On the other hand, *NTPCR* is a non-specific nucleoside triphosphatase related to pyrimidine deoxyribonucleotide biosynthesis from CTP and pyrimidine metabolism pathways. *NTPCR*-associated disorders include vasculogenic impotence and western equine, whereas phenotypes from GWAS related to HPP involve bone density (rs149310507) and body mass index (rs12035585) [21,22]. HPO data were not available for *NTPCR*.

Additional direct and via partners PPI were noted among *BGLAP* (Bone Gamma-Carboxyglutamate Protein), *RUNX2* (RUNX Family Transcription Factor 2), *ENPP1* (Ectonucleotide Pyrophosphatase/Phosphodiesterase 1), and *ALPL* interacting in the endochondral ossification with skeletal dysplasias, endochondral ossification, OSX and miRNAs in tooth development, and FGF23 signaling in hypophosphatemic rickets and related disorders pathways (Figure 1).

*BGLAP* acts as a regulator of bone remodeling and as a hormone secreted by osteoblasts, which regulates different cellular processes, such as energy metabolism, male fertility, and brain development. Diseases associated with *BGLAP* include osteitis fibrosa, renal osteodystrophy, and osteoporosis, whereas phenotypes from GWAS related to HPP comprise heel bone mineral density (rs116251020) [21,22]; HPO data were not available for *BGLAP*.

*RUNX2* is a transcription factor essential for osteoblastic differentiation and skeletal morphogenesis and acts as a scaffold for nucleic acids and regulatory factors involved in skeletal gene expression. Diseases related to *RUNX2* involve cleidocranial dysplasia and metaphyseal dysplasia with maxillary hypoplasia with or without brachydactyly [21]. Functional annotation and HPO implication of *RUNX2* related to HPP involve bone density (rs111469485, rs111928541, rs113308638, rs115695595, rs12201899, rs13437231, rs142025540, rs183798784, rs184065563, rs191485704, rs549531306, rs56078331, rs565953603, rs74579060, rs74587175, rs750754420, rs767861798, rs79608764, rs9472482, rs9472489), heel bone mineral density (rs11442031, rs1283935, rs491616, rs575445, rs658710) [21,22], hip dislocation, short stature, osteoporosis of vertebrae, brachydactyly, platyspondyly, metaphyseal dysplasia, skeletal dysplasia, wormian bones, scoliosis, recurrent fractures, kyphosis, osteoporosis, recurrent respiratory infections, coxa vara, genu valgum, decreased skull ossification, premature loss of teeth, enamel hypoplasia, and spondylolisthesis [21,23].

*ENPP1* is a member of the ectonucleotide pyrophosphatase/phosphodiesterase family with functions in bone mineralization and soft tissue calcification by regulating pyrophosphate levels. Diseases associated with *ENPP1* include Cole disease, autosomal recessive hypophosphatemic rickets, type 2 diabetes mellitus, HPP, and autism spectrum disorder [21]. The functional annotation and HPO implication of *ENPP1* related to HPP encompass chondrocalcinosis (rs766592), alkaline phosphatase measurement (rs453639), body mass index (rs3756784), body mass index-adjusted waist-hip ratio (rs4470875) [21,22], growth delay, short stature, seizure, skeletal dysplasia, coxa vara and valga, genu valgum and varum, osteomalacia, delayed skeletal maturation, tibial bowing, craniosynosostosis, hypoplasia of teeth, delayed eruption of teeth, carious teeth, nephrocalcinosis, blue sclerae, ankylosis, and elevated alkaline phosphatase of bone origin [21,23].

Limitations

The findings in this report should be cautiously interpreted because of potential limitations. This retrospective analysis included a small number of cases, which may limit the generalizability of our findings. Yet, some similarities were observed, including intrafamilial and interfamilial variability of phenotypes with an identical biallelic *ALPL* mutation c.1001G>A and c.571G>A [4,6,9], as well as heterogeneity through our systematic review of the literature. Nevertheless, evidence inferred from in silico predictions provides valuable insights into the genetic composition of the studied cases. The latter implies a restricted genetic scope. While this targeted approach enabled an in-depth analysis of specific variants, it did not reflect the entire spectrum of possible genetic risk factors for HPP.

Additional limitations include the assessments regarding HPP progression and limited environmental data. Detailed information on developmental health, lifestyle, diet, or noxious exposures (e.g., drugs, adverse maternal conditions, toxins) was not systematically available for all family members, which limits our ability to explore the gene-environment interactions on HPP risk. Furthermore, the lack of full gene sequencing and potentially deletion/duplication analysis for *ALPL* between the unaffected family members may have masked unique genetic factors within the nuclear families. Lastly, findings from bioinformatic algorithms can only be taken as suggestive evidence in the absence of functional validation.

## Conclusions

Our report identified 17 variants located in the exome and four classified as non-coding by employing in silico analyses for *ALPL* (rs121918007 and rs121918009). The dual effect influencing regions of the alternative transcripts of the gene revealed associations with childhood, infantile, and odontohypophosphatasia presentations, as well as serum alkaline phosphatase levels, choline phosphate levels, osteogenesis imperfecta, and inborn genetic diseases, which suggest a complex genetic etiology for HPP.

Based on gene network modeling, we found 10 intersecting genes (*PTS, NBPF3, SLC30A7, TPK1, NTPCR, BGLAP, RUNX2, ENPP1, SLC30A6, *and* GCH1*) interacting with *ALPL* and highlighting their potential impact on bone formation, homeostasis, and gene expression, potentially affecting pathways involved in skeletal system development, metabolism, remodeling DNA, transcriptional factors and regulators, and ionic homeostasis, enhancing our ability to understand biological mechanisms contributing to HPP development. Finally, the accrued evidence on these *ALPL* variants provides valuable insights for healthcare providers and genetic counseling. However, further molecular and mechanistic investigations are needed to validate the functional impact of these variants on HPP development.
